# Chopper Is Prodeath Regardless of the Effect of p75ICD on Sensitivity to Oxidative Stress

**DOI:** 10.1155/2011/391659

**Published:** 2011-09-05

**Authors:** Alliya Qazi, Marc W. Halterman, Zhiping Mi, Tong Zhang, Nina F. Schor

**Affiliations:** ^1^Department of Pediatrics, Golisano Children's Hospital at URMC, University of Rochester Medical Center, Rochester, NY 14642, USA; ^2^Department of Neurology, University of Rochester Medical Center, Rochester, NY 14642, USA; ^3^The Center for Neural Development and Disease, University of Rochester Medical Center, Rochester, NY 14642, USA; ^4^Departments of Neurobiology & Anatomy, University of Rochester Medical Center, Rochester, NY 14642, USA

## Abstract

*Background*. The intracellular domain (ICD) of the neurotrophin receptor, p75NTR, exhibits variably pro- and antiapoptotic activity and has been implicated in neurodegenerative and neurodestructive disease. The molecular determinants of these cellular effects are not completely understood. The “Chopper” domain of p75ICD has been shown to be proapoptotic in *in vitro* systems in which p75ICD is proapoptotic. The effects of Chopper in systems in which p75ICD is antiapoptotic and, therefore, whether or not Chopper accounts for the variability of the cellular effects of p75ICD are not known. We therefore examined the effects of deletion of Chopper on the effects of p75ICD on *in vitro* cell culture systems in which p75ICD is pro- or antiapoptotic, respectively. *Results*. In HN33.11 murine neuroblastoma-hippocampal neuron hybrid cells, p75ICD is antiapoptotic. In NIH 3T3 cells, p75ICD is proapoptotic. In both cell lines deletion of the Chopper domain from p75ICD decreases the incidence of apoptosis resulting from oxidative stress. Thus, irrespective of the nature of the effects of p75ICD on the cell, its Chopper domain is proapoptotic. *Conclusions*. Expression of p75ICD can enhance or attenuate oxidative induction of apoptosis. Variability of the effects of p75ICD is not related to variability of the effects of its Chopper domain.

## 1. Background

The neurotrophin receptor, p75NTR, has been implicated in the pathogenesis of neurodegenerative and neurodestructive diseases, including Alzheimer's disease [1–3], Parkinson's disease [4], invasive glioblastoma [5], and chemoresistant neuroblastoma [6]. It can be pro- or antiapoptotic depending on the milieu in which it is expressed [6, 7]. Its activity appears to be mediated by its intracellular domain (p75ICD; [8, 9]). p75ICD can substitute for full-length p75NTR in both protective [8, 9] and death-inducing [7] models.

p75NTR is a member of the tumor necrosis factor family of death receptors. As such, its C-terminal, CD95-like death domain was originally assumed to be responsible for its proapoptotic properties. However, the p75NTR death domain does not behave like the death domains of other tumor necrosis factor family death receptors [10–12]. The Chopper region at the juxtamembrane end of p75ICD has been shown to be both necessary and sufficient for apoptosis induction by p75NTR. Chopper must be either membrane-anchored or palmitoylated to function in this way; in fact, when it is not, it is a dominant negative influence on this activity in models in which p75NTR is proapoptotic [13].

We have characterized neural crest tumor models in which p75ICD is either protective against oxidative stress and, therefore, antiapoptotic [8, 9] or facilitatory of mitotic arrest and, therefore, proapoptotic [7]. We now demonstrate that, in both antiapoptotic and proapoptotic model systems, p75ICD-bound Chopper appears to be proapoptotic. This suggests that the variable nature of the activity of p75ICD vis-à-vis cell survival is not the result of variability of Chopper function.

## 2. Methods

### 2.1. Genetic Constructs and Cell Culture Models

The HN33.11 line (gift from Bruce Wainer, Emory University, Atlanta, GA) is a hybrid line of murine neuroblastoma and primary hippocampal neurons. These cells have been used as an immortalized model for the study of disorders of the hippocampus [14], one of the few brain regions in which p75NTR is expressed in the adult [15]. HN33.11 cells were cultured in Dulbecco's Minimal Essential Medium (DMEM) supplemented with 8% fetal bovine serum (Atlanta Biologicals, Norcross, GA). 

**Figure 1:**
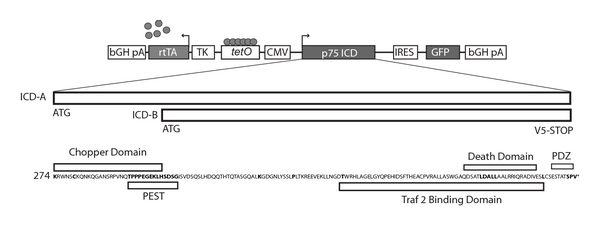
Regulating p75ICD expression using the pBIG2i Tet-inducible expression system. Full-length p75ICD (ICD-A) or the N-terminal deletion mutant (ICD-B) was expressed from the bidirectional tetracycline regulated vector pBIG2i. In the presence of ligand (doxycycline), the reverse transactivator (rtTA) expressed from the thymidine kinase promoter (TK) binds to the heptemerized tetO sequence activating transgene expression from both the minimal TK and CMV promoters. Inclusion of the IRES-eGFP cassette facilitates the analysis of p75ICD expressing clones. Domains included in the full-length ICD include the amino terminal “chopper domain”, tandem PEST sequence, Traf2- and PDZ-binding domains, and the carboxyl terminal death domain. In addition to using IRES-mediated GFP expression, both ICD expression constructs contain the V5 epitope to monitor inducible ICD transgene expression.

Full-length p75ICD (A) and Chopper-deleted p75ICD (B) constructs were cloned with a V5 epitope into the pBig2i-MCS-GFP doxycycline- (Dox-; Sigma-Aldrich, St. Louis, MO) regulated vector (Invitrogen, Carlsbad, CA; Figure 1). Plasmids were transfected into HN33.11 cells using Lipofectamine 2000 (Invitrogen) and stable lines were selected for resistance to Hygromycin (400 mg/mL; Invitrogen). Construct inducibility was verified by fluorescence microscopy for GFP expression and Western blot analysis for p75ICD fragment expression after Dox exposure for 36–48 hours. Individual GFP-positive and GFP-negative clones were isolated for study.

NIH 3T3 cells (American Type Culture Collection, Rockville, MD) were also studied to determine the effects of transfection with an expression construct for (A) or (B), as, unlike HN33.11 cells, they do not express endogenous p75NTR (Mi and Schor, unpublished results; also see Figure 8, mcs lane). Transfection, selection, and clonal isolation of NIH 3T3 cells were performed as described above for HN33.11 cells.

### 2.2. Western Blots

Protein concentrations were determined using the Bradford assay (Bio-Rad Protein Assay, Bio-Rad Laboratories, Hercules, CA), and 15 *μ*g of protein was loaded onto a 15% sodium dodecyl sulfate-polyacrylamide gel and transferred to a polyvinylidine fluoride membrane (Millipore, Bedford, MA) as we have described previously [16]. Western blot analysis was performed using anti-p75ICD (Promega, Fitchburg, WI; 1 : 1000) and anti-V5 (Novus, Littleton, CO; 1 : 4000) antibodies.

### 2.3. Exposure to Oxidant Stress

Cells were treated with 6-hydroxydopamine (6-OHDA; Sigma-Aldrich) or H_2_O_2_ in complete (i.e., serum-containing) culture medium for 1 hour. Media were replaced and cells were incubated for 48 hours before analysis via flow cytometry or the MTS viability assay (see what follows).

### 2.4. Cell Cycle Analysis

2 × 10^6^ cells were fixed in ethanol, treated with RNase, and stained with propidium iodide to detect DNA. GFP-expressing and -nonexpressing cells were analyzed as we have previously described [6] in the presence and absence of Dox. 

### 2.5. MTS Assay

20 *μ*L of CellTiter 96 AQueous One Solution Reagent (Promega) was added into each well of the 96-well assay plate containing the samples in 100 *μ*L of culture medium. The plate was incubated at 37°C for 1 hour in a humidified, 5% CO_2_ atmosphere before reading the optical density of each well at 490 nm (OD_490 nm_) in a plate reader.

### 2.6. Determination of Caspase-3 Cleavage by Western Blot

Cells were harvested at 0, 6, 9, 12, and 24 hours after 6-OHDA or vehicle treatment. Cell pellets were resuspended into radioimmunoprecipitation assay buffer (10 mM Tris, pH 8; 150 mM NaCl; 0.1% Nonidet P-40; 0.5% sodium deoxycholate; 0.1% SDS; 1mM phenylmethylsulfonyl fluoride; 4 *μ*g/mL aprotinin; and 1 mM sodium orthovanadate; all from Sigma-Aldrich) and, after sonication and centrifugation, aliquots of supernatants containing equivalent amounts of protein were loaded onto a sodium dodecyl sulfate-polyacrylamide gel for Western blotting. Blots were stained with a primary rabbit antibody against cleaved caspase-3 and a goat-anti-rabbit secondary antibody (Cell Signaling Technology, Beverly, MA).

### 2.7. Hoechst Dye Assay for Apoptosis

After treatment with 6-OHDA, cells were washed once with Dulbecco's phosphate-buffered saline and fixed with cold 95% ethanol. Hoechst dye 33342 (Sigma-Aldrich; 2 mg/mL stock solution) was then added into the cells in a 1 : 200 dilution in Dulbecco's phosphate-buffered saline; the cells were then incubated for 30 minutes at room temperature. Cells were washed once in Dulbecco's phosphate-buffered saline and mounted on slides for fluorescence microscopic analysis.

### 2.8. Statistical Analysis

Between-group comparisons for parametric variables were performed using Student's *t-*test. Differences were considered statistically significant at the *P* ≤ 0.05 level.

## 3. Results

### 3.1. Analysis of the Dox-Regulated p75ICD Vector System in HN33.11 Cells

**Figure 2:**
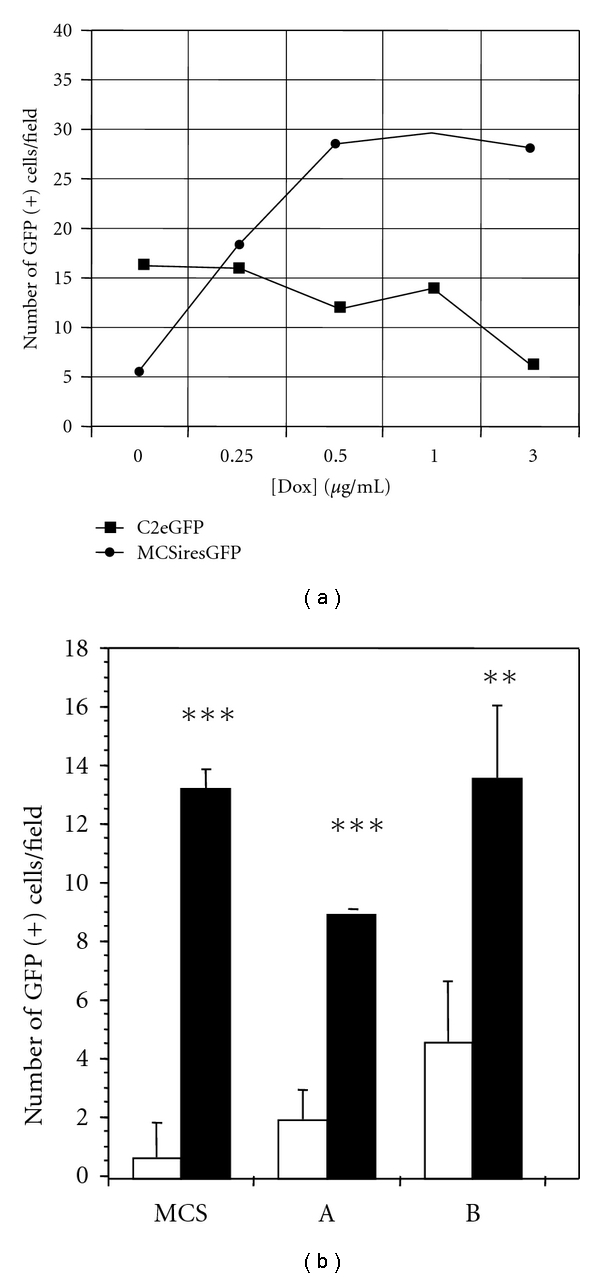
(a) Results of transfection of HN33.11 cells with green fluorescent protein- (GFP-) linked empty plasmid (C2eGFP) or GFP-linked multiple cloning site in the same plasmid with a doxycycline- (Dox-) inducible internal ribosome entry site (MCSiresGFP). Dox elicits production of GFP in the MCSiresGFP-transfected cells, but not in C2eGFP-transfected cells. (b) Dox-inducibility of MCSiresGFP-linked expression constructs for (A) and (B) as defined in Figure 1. Open bars: cells maintained in the absence of Dox; closed bars: cells maintained in the presence of Dox (1 *μ*g/mL). Results shown are the mean ± SEM of three independent experiments performed. ***P < 0.001; **P < 0.01 relative to absence of Dox, Student's *t*-test.

HN33.11 cells transfected with either the control GFP vector, C2eGFP, or the test plasmid, MCSiresGFP, were treated with Dox (0.5–3 *μ*g/mL). Dox induced GFP expression in MCSiresGFP-transfected cells. In contrast, Dox treatment did not enhance GFP expression in C2eGFP-transfected cells (Figure 2(a)).

HN33.11 cells were then transfected with either the MCSiresGFP, (A)-GFP, or (B)-GFP expression construct. Dox induced GFP expression in all cases, although the (A) and (B) constructs exhibited some degree of “leakiness” in the absence of Dox (Figure 2(b)). 

**Figure 3:**
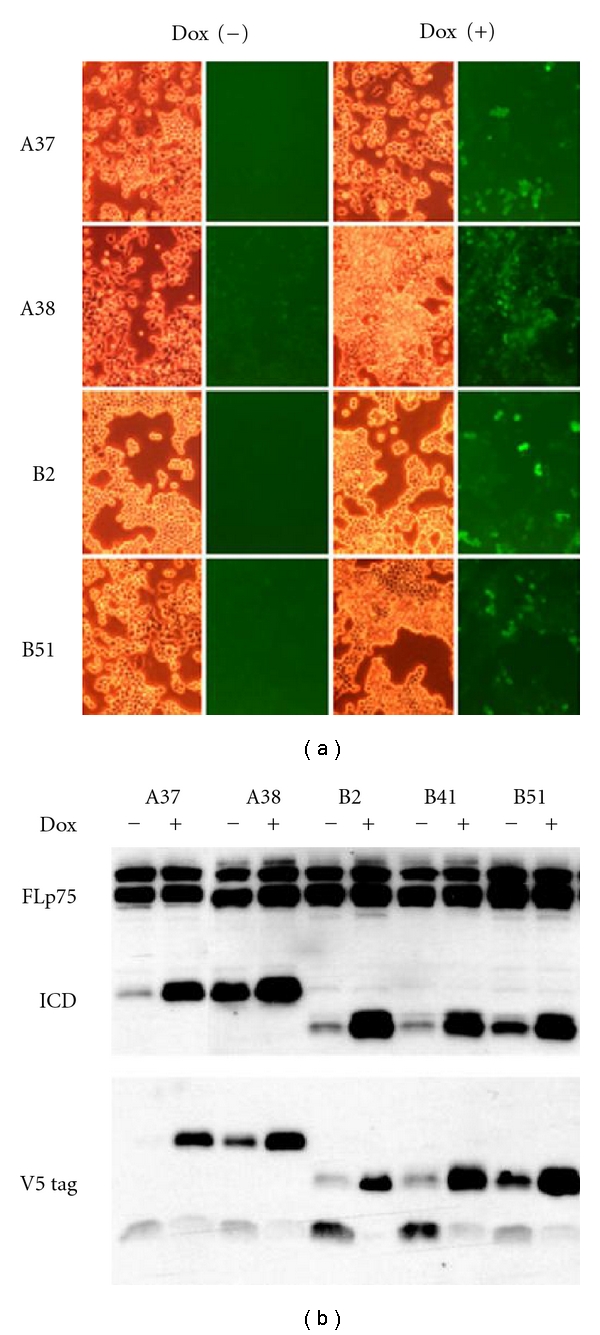
(a) Light and fluorescent micrographs of stable HN33.11 cells expressing (A)-GFP or (B)-GFP, respectively. Results from two stable clones of each transfection pool are shown. Cells are treated with vehicle (Dox−) or 1 *μ*g/mL Dox (Dox+). (b) Western blot of homogenates of stable clones showing endogenous production of full-length p75NTR (FLp75), Dox-induced, but “leaky” production of (A) and (B) and of the V5 tag. Control values for each clone do not differ significantly between GFP(−) and GFP(+) cells (Student's *t*-test). ***P < 0.001 relative to GFP(−) cells.

Stable lines were created from two clones each of (A)-GFP-transfected and (B)-GFP-transfected cells. Dox-induced GFP expression was demonstrated in all cases (Figure 3(a)) and GFP-positive and GFP-negative clones were isolated. Figure 3(b) demonstrates, however, that these HN33.11-derived clones also express endogenous full-length p75NTR at approximately the same level for all of the clones, making the induced p75ICD (A) and (B) a presumed increment to the p75ICD that could be generated through endogenous cleavage of p75NTR.

### 3.2. Protective Effects of (A)-GFP Expression in HN33.11 Cells

Our previous studies demonstrated the protective effects of transfection with a p75ICD expression construct against oxidative stress in p75NTR-negative PC12 pheochromocytoma cells [8, 9]. Given that transfection of HN33.11 cells with (A)-GFP puts p75ICD into cells that are already p75NTR-positive, we sought first to test the hypothesis that (A)-GFP expression would enhance the resistance of HN33.11 cells to oxidant stress. As HN33.11 cells are known to express a dopamine uptake system [17], we used 6-OHDA to inflict oxidative stress, as we have in our previous publication [8].

**Figure 4:**
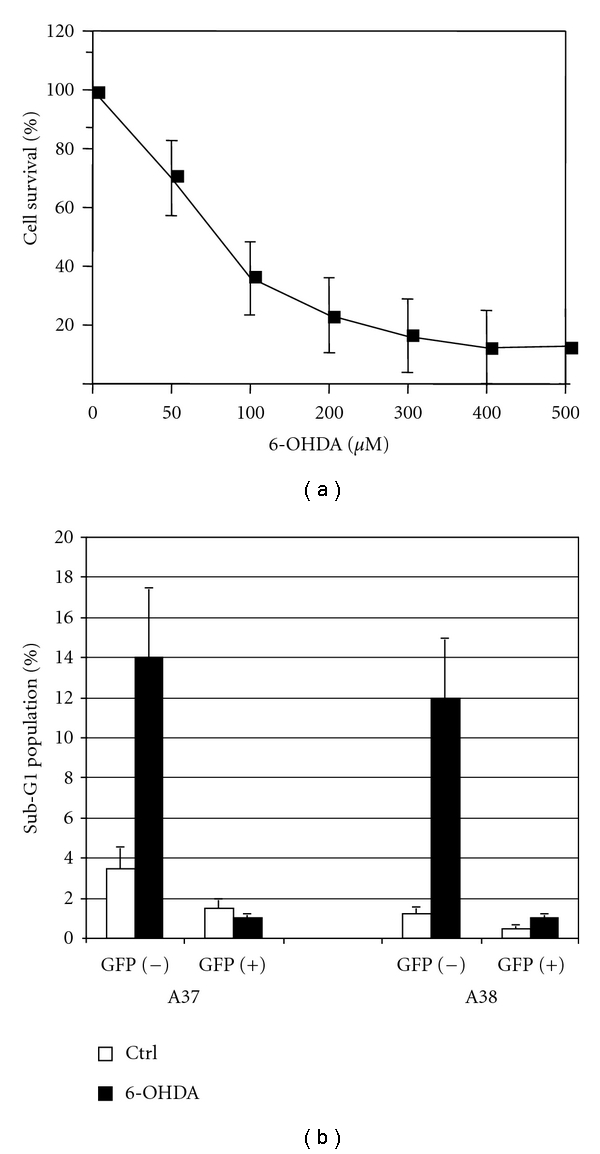
(a) Concentration-survival curve of HN33.11 cells to 6-hydroxydopamine (6-OHDA). Cell survival was determined by MTS assay as described in Methods. (b) Effects of Dox-induced expression of (A)-GFP on induction cell death by 6-OHDA in HN33.11 cells (clones A37 and A38). The stable lines indicated were plated at 5×10^5^ cells per 60 mm dish and exposed to 6-OHDA (300 *μ*M) for 1 hour and incubated for 48 hours prior to analysis by flow cytometric quantitation of sub-G1 nuclei. The results of one experiment of three performed are shown.

Figure 4(a) shows the concentration-cell survival curve of HN33.11 cells to 6-OHDA. The stable lines indicated were plated at 5 × 10^5^ cells per 60 mm dish and exposed to 6-OHDA (300 *μ*M) for 1 hour and incubated for 48 hours prior to analysis by flow cytometric quantitation of sub-G1 (i.e., apoptotic) nuclei. Dox-induced expression of (A)-GFP in HN33.11 cells (clones A37 and A38) is antiapoptotic (Figure 4(b)).

To ensure that transfection alone with construct (A) or construct (B) with or without GFP did not alter progression of HN33.11 cells through the cell cycle, we examined the transfectants by flow cytometry in the absence of 6-OHDA. The data shown in Table 1 indicate that neither construct (A) nor construct (B) in the native or GFP-linked state alters the cell cycle distribution of HN33.11 cells.

### 3.3. Effects of Removal of the Chopper Domain from (A)-GFP on Protection from Oxidative Stress in HN33.11 Cells

Expression construct (B)-GFP produces a GFP-linked protein that represents p75ICD minus the Chopper domain. Two clones (B2 and B51), both stable transfectants, of (B)-GFP-transfected HN33.11 cells were used to test the effects of deletion of Chopper on the protective effects of (A)-GFP (clones A37 and A38).

**Figure 5:**
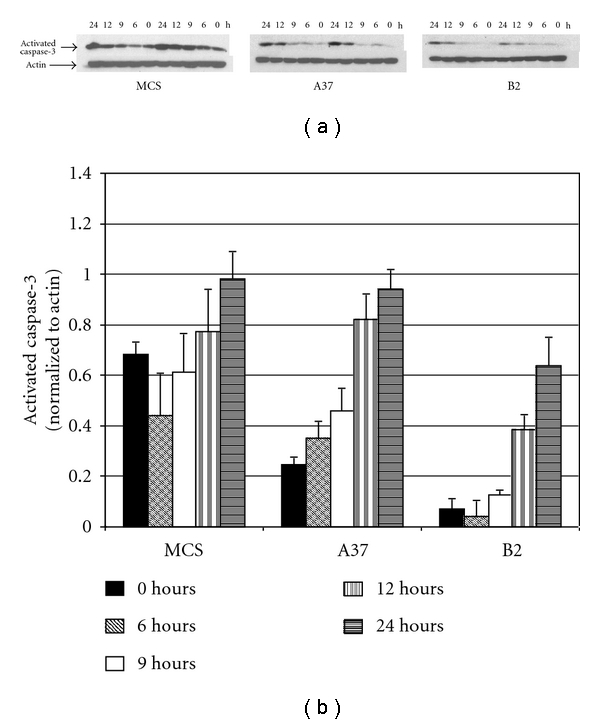
Time course of activation of caspase-3 after treatment with 6-OHDA (150 *μ*M, 1 hour exposure) in stable MCS, A37, and B2 clones. Activated caspase-3 levels were determined by Western blotting and optical densitometry of the resulting bands. Protection from caspase-3 activation is greatest in B2 cells and less robust in A37 cells.

Results for clones A37 and A38 were comparable, as were results for clones B2 and B51. Data are therefore shown for clones A37 and B2.  Figure 5 compares the time course for detection of activated caspase-3 after a 1 hour exposure to 6-OHDA (150 *μ*M) in MCS-GFP-transfected (control) cells and clones A37 and B2. As expected, transfection with (A)-GFP protects HN33.11 from 6-OHDA-induced activation of caspase-3 and attenuates the time course of such activation. Transfection with (B)-GFP makes this effect more pronounced; that is, deletion of the Chopper domain further protects the cells from 6-OHDA and attenuates the time course of caspase-3 activation.

**Figure 6:**
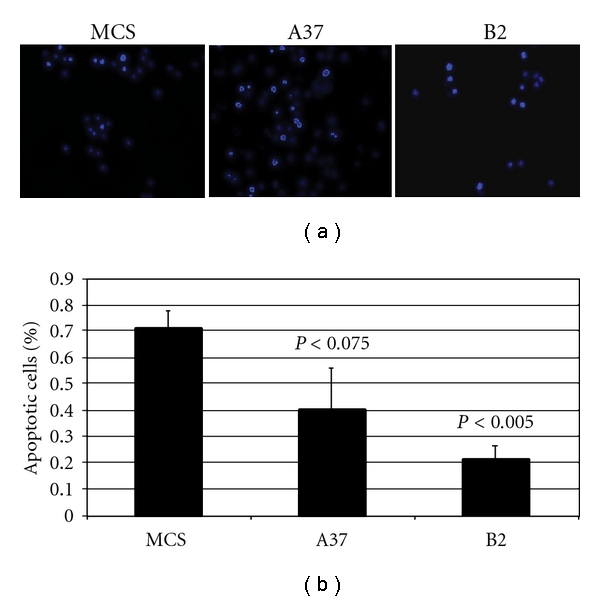
Hoechst dye staining of MCS, A37, and B2 cells 48 hours after treatment with 6-OHDA (150 *μ*M, 1 hour exposure). Two-hundred total cells were counted in each treated well to determine the percent of the cells that exhibited nuclear condensation and/or fragmentation and margination of DNA. *P* values for A37 and B2 cells are calculated by Student's *t*-test relative to MCS cells. Protection from morphologic apoptosis is greatest in B2 cells and less robust in A37 cells.

Morphological studies (Figure 6) document the attenuation of apoptosis induction by 6-OHDA (150 *μ*M for 1 hour; photos taken 24 hours after completion of 6-OHDA treatment) in clone A37 relative to stable MCS-GFP cells and in clone B2 relative to clone A37. 

**Figure 7:**
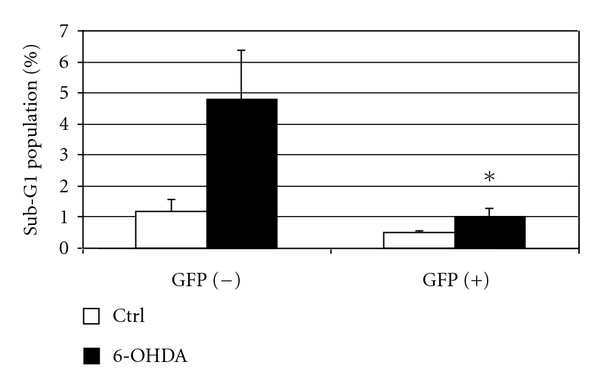
Stable (B)-GFP transfectants were treated with vehicle or 6-OHDA in the absence or presence of Dox and studied by flow cytometry as for (A)-GFP transfectants shown in Figure 4. The results of one experiment of two performed are shown. Control values are not siginificantly different (Student's *t*-test) between GFP(−) and GFP(+) cells. *P = 0.05 relative to GFP(−) cells.

Flow cytometric analysis of the prevalence of sub-G1 nuclei cells of clone B2 treated with 6-OHDA (300 *μ*M) is shown in Figure 7. Comparison of these results with those shown for the (A)-GFP clones in Figure 4(b) demonstrates that removal of the Chopper domain from the (A)-GFP construct does not block the protection of HN33.11 cells from oxidative stress.

### 3.4. Analysis of the Dox-Regulated p75ICD Vector System in NIH 3T3 Cells

In an effort to study the effects of transfection with (A)-GFP and (B)-GFP on cells that do not endogenously express p75NTR or p75ICD, we made stable transfectants of NIH 3T3 cells with these constructs and with MCS-GFP (control). Because NIH 3T3 cells do not express a catecholamine transporter [18], we subjected them to oxidative stress using H_2_O_2_. Our previous studies had demonstrated the qualitative equivalency of 6-OHDA and H_2_O_2_ as generators of oxidative stress in other cellular models of p75NTR-mediated modulation of susceptibility to reactive oxygen species [8].

**Figure 8:**
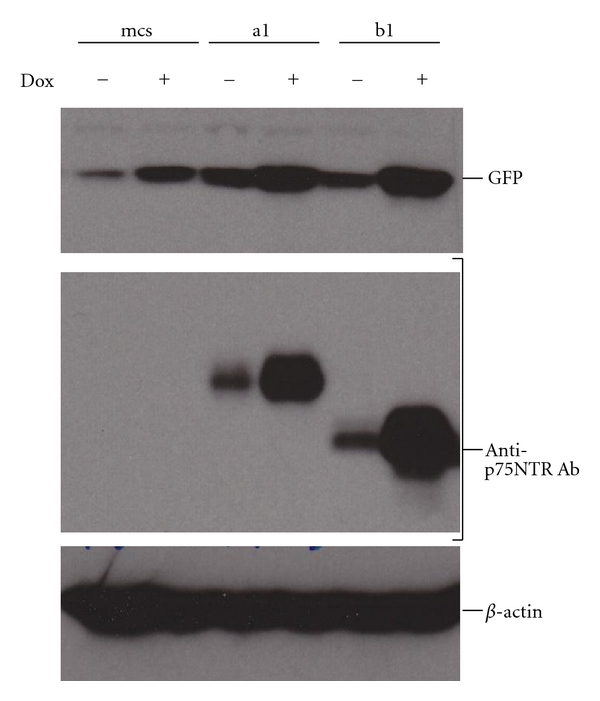
Induction by Dox is shown for GFP in all cell lines, for (A)-GFP in the a1 clone, and for (B)-GFP in the b1 clone of NIH 3T3 cells by Western blotting as described in Methods. Note that some (A)-GFP and (B)-GFP are produced in a1 and b1 clones, respectively, in the absence of Dox.

Figure 8 demonstrates that NIH 3T3 cells transfected with MCS do not express p75ICD; transfection with construct (A) or construct (B), respectively, results in expression of each of the two fragments of p75ICD. In this context, the expression construct is sufficiently “leaky” as to preclude turning expression on and off using Dox. Effects of oxidative stress on NIH 3T3 transfectants are therefore the same in the presence and absence of Dox (data not shown).

### 3.5. Effects of Expression of Construct (A) or Construct (B) on Cell Death Induced by Oxidative Stress in NIH 3T3 Cells

**Figure 9:**
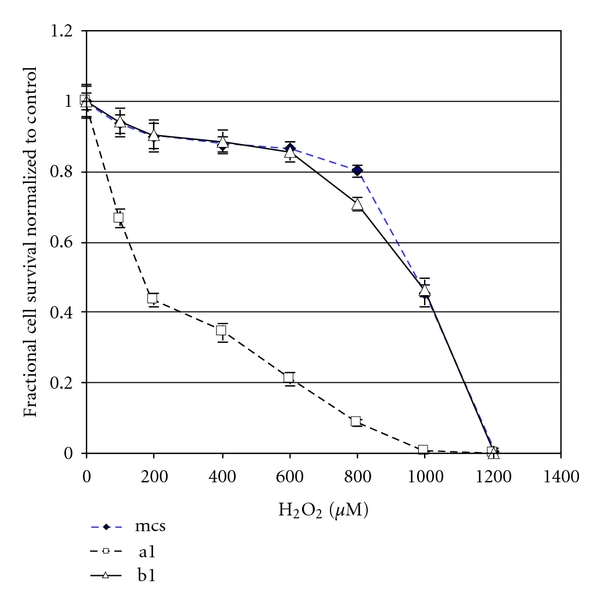
p75ICD ((A)-GFP; a1 clone) potentiates induction of cell death (MTS assay) by H_2_O_2_ in NIH 3T3 cells. Deletion of the Chopper domain from p75ICD ((B)-GFP; b1 clone) restores the resistance of these cells to H_2_O_2_. Results shown are the mean ± SEM of three independent experiments performed.

In contrast to the protective effects of construct (A) in HN33.11, construct (A) potentiates cell death induced by treatment with H_2_O_2_ in NIH 3T3 cells (Figure 9). Deletion of the Chopper domain and transfection with the resultant construct (B) returns the response of NIH 3T3 cells to oxidative stress to that of the MCS-transfected cells; that is, deletion of the Chopper domain protects NIH 3T3 cells from the effects of transfection with p75ICD. This suggests that, although p75ICD expression has opposite effects on sensitivity to oxidative stress in HN33.11 and NIH 3T3 cells, respectively, the Chopper domain is proapoptotic in both cell lines and the differential effects of p75ICD (i.e., construct (A)) are not related to differential signaling through Chopper.

## 4. Discussion

The neurotrophin receptor, p75NTR, functions both as a low affinity, independent receptor and as a coreceptor with TrkA. In its independent function, it serves as an initiator of both pro- and antiapoptotic signaling pathways. It is one of a growing family of “dependence receptors”—receptors that confer cellular dependence on growth factors for life-or-death decision-making [19].

The binding of nerve growth factor to the extracellular domain of p75NTR and the subsequent binding of a variety of interactor proteins with its intracellular domain result in the sequential cleavage of p75NTR by *α*- and *γ*-secretase to liberate the intracellular domain of p75NTR, p75ICD [20]. We have previously demonstrated that p75ICD is sufficient for the variably pro- and antiapoptotic activities of p75NTR in some *in vitro* systems [8, 9]. An N-terminal segment of p75ICD has been termed “Chopper” and has been shown to be proapoptotic in an *in vitro* system in which holo-p75NTR is proapoptotic [13]. In the present study, we asked the question of whether Chopper would, conversely, be antiapoptotic in an *in vitro* system in which holo-p75NTR is antiapoptotic. That is, we tested the hypothesis that Chopper activity determines whether p75NTR is pro- or antiapoptotic in a given system.

The results we present are not consistent with this hypothesis. They demonstrate that Chopper is proapoptotic whether holo-p75NTR is pro- or antiapoptotic. The variability of the effects of p75NTR on cell survival is therefore not related to variability of the effects of Chopper.

While the tyrosine kinase receptors have long been known to modulate such processes as cell death, proliferation, protection, and differentiation in development and disease of the nervous system (reviewed in [21]), the involvement of p75NTR in development and disease is much less well characterized (reviewed in [22]). However, recent studies make it likely that p75NTR plays different roles in Alzheimer's disease [1–3], Parkinson's disease [4], differentiation and synaptic connectivity along the neuraxis [23], resistance of neural tumors to chemotherapy [6], and migration of glioblastoma cells away from the primary tumor [5].

Expression of p75NTR in the central nervous system is developmentally restricted such that, as humans progress from embryonic life to adulthood, p75NTR goes from ubiquitous expression to expression largely limited to cholinergic neurons in the basal forebrain and hippocampus (reviewed in [22]). In addition, p75NTR and amyloid precursor protein (APP) have been shown to interact and, in their interaction, to induce neuronal death. p75NTR influences processing of APP and both NGF and *β*-amyloid alter the p75NTR-APP interaction [24]. Furthermore, p75NTR is a substrate for presenilin-1 and the two are coregulated in model systems and during normal development [1, 2]. These observations have implicated p75NTR in the pathogenesis of Alzheimer's disease.

The proNGF-sortillin-p75NTR complex has been implicated in apoptosis associated with Parkinson's disease. This complex has been suggested as a potential target in the prevention of degeneration of neurons in the substantia nigra [4].

Pruning of axons during developmental neurite outgrowth has recently been shown to be a p75NTR-dependent process [25]. Brain-derived neurotrophic factor (BDNF) is the cognate p75NTR ligand that orchestrates activity-dependent persistence of axons during development. Those axons that are less active degenerate in a process that requires p75NTR.

Resistance of PC12 pheochromocytoma cells to chemotherapy [6], potentiation of neuroblastoma cell death after oxidative chemotherapy [26], and migration away from the primary tumor with central nervous system invasiveness of glioblastomas [5] all depend critically on p75NTR.

Understanding the structural and molecular underpinnings of p75NTR function and its context dependence may therefore identify novel targets for the treatment or prevention of neurodegenerative disease, developmental aberrations of the nervous system, and nervous system cancer. Molecular “dissection” of p75ICD may facilitate functional segregation of its pro- and antiapoptotic effects and facilitate manipulation of one or the other of the signaling cascades triggered by p75NTR and involved in specific pathologic or developmental processes. The results of the present study raise the question of whether Chopper-free fragments of p75NTR or p75ICD might be prototypes for antioxidant and antiapoptotic therapeutic strategies in the nervous system.

## 5. Conclusions

The cellular function of p75NTR is cell- and context-dependent. In contrast, the cellular function of Chopper is not dependent on the function of p75NTR within a particular cell or context. Chopper is proapoptotic in cells in which p75NTR is either pro- or antiapoptotic. The variability of function of p75NTR is, therefore, not a result of variable function of Chopper. Furthermore, Chopper-free fragments of p75NTR or p75ICD may be viable prototypes for the development of antiapoptotic strategies for the treatment of neurodegenerative disease.

## Figures and Tables

**Table 1:** Baseline influence of p75ICD fragment expression on cell cycle.

	Construct A		Construct B	
	eGFP−	eGFP+	eGFP−	eGFP+
Sub-G1	1.9	2.3	3.8	6.6
G1	66.6	41.2	62.5	50.0
S	18.6	28.2	19.3	22.1
G2	11.5	26.6	12.9	18.9

## References

[1] Mirnics ZK, Mirnics K, Terrano D, Lewis DA, Sisodia SS, Schor NF (2003). DNA microarray profiling of developing PS1-deficient mouse brain reveals complex and coregulated expression changes. *Molecular Psychiatry*.

[2] Mirnics K, Mirnics ZK, Arion D (2005). Presenilin-1-dependent transcriptome changes. *Journal of Neuroscience*.

[3] Coulson EJ, May LM, Sykes AM, Hamlin AS (2009). The role of the p75 neurotrophin receptor in cholinergic dysfunction in Alzheimer's disease. *Neuroscientist*.

[4] Chen LW, Yung KKL, Chan YS, Shum DKY, Bolam JP (2008). The proNGF-p75NTR-sortilin signalling complex as new target for the therapeutic treatment of Parkinson's disease. *CNS and Neurological Disorders*.

[5] Johnston ALM, Lun X, Rahn JJ (2007). The p75 neurotrophin receptor is a central regulator of glioma invasion. *PLoS Biology*.

[6] Yan C, Liang Y, Nylander KD (2002). p75-nerve growth factor as an antiapoptotic complex: independence versus cooperativity. *Molecular Pharmacology*.

[7] Yan C, Mirnics ZK, Portugal CF (2005). Cholesterol biosynthesis and the pro-apoptotic effects of the p75 nerve growth factor receptor. *Molecular Brain Research*.

[8] Tyurina YY, Nylander KD, Mirnics ZK (2005). The intracellular domain of p75NTR as a determinant of cellular reducing potential and response to oxidant stress. *Aging Cell*.

[9] Mi Z, Rogers DA, Mirnics ZK, Schor NF (2009). P75NTR-dependent modulation of cellular handling of reactive oxygen species. *Journal of Neurochemistry*.

[10] Liepinsh E, Ilag LL, Otting G, Ibáñez CF (1997). NMR structure of the death domain of the p75 neurotrophin receptor. *EMBO Journal*.

[11] Kong H, Kim AH, Orlinick JR, Chao MV (1999). A comparison of the cytoplasmic domains of the Fas receptor and the p75 neurotrophin receptor. *Cell Death and Differentiation*.

[12] Coulson EJ, Reid K, Barrett GL, Bartlett PF (1999). p75 neurotrophin receptor-mediated neuronal death is promoted by Bcl-2 and prevented by Bcl-x(L). *Journal of Biological Chemistry*.

[13] Coulson EJ, Reid K, Baca M (2000). Chopper, a new death domain of the p75 neurotrophin receptor that mediates rapid neuronal cell death. *Journal of Biological Chemistry*.

[14] Lee HJ, Hammond DN, Large TH (1990). Neuronal properties and trophic activities of immortalized hippocampal cells from embryonic and young adult mice. *Journal of Neuroscience*.

[15] Korade Z, Mi Z, Schor NF (2007). Expression and p75 neurotrophin receptor dependence of cholesterol synthetic enzymes in adult mouse brain. *Neurobiology of Aging*.

[16] Halterman MW, De Jesus C, Rempe DA, Schor NF, Federoff HJ (2008). Loss of c/EBP-*β* activity promotes the adaptive to apoptotic switch in hypoxic cortical neurons. *Molecular and Cellular Neuroscience*.

[17] Jin LQ, Cai G, Wang HY, Smith C, Friedman E (1998). Characterization of the phosphoinositide-linked dopamine receptor in a mouse hippocampal-neuroblastoma hybrid cell line. *Journal of Neurochemistry*.

[18] Wikigenes http://www.wikigenes.org/?search=catecholamine+transporter+fibroblasts&db=_any&cat=&type=&field=&org=&action=go&ftype=0.

[19] Mehlen P, Bredesen DE (2004). The dependence receptor hypothesis. *Apoptosis*.

[20] Kanning KC, Hudson M, Amieux PS, Wiley JC, Bothwell M, Schecterson LC (2003). Proteolytic processing of the p75 neurotrophin receptor and two homologs generates C-terminal fragments with signaling capability. *Journal of Neuroscience*.

[21] Skaper SD (2008). The biology of neurotrophins, signalling pathways, and functional peptide mimetics of neurotrophins and their receptors. *CNS and Neurological Disorders*.

[22] Schor NF (2005). The p75 neurotrophin receptor in human development and disease. *Progress in Neurobiology*.

[23] Bartkowska K, Turlejski K, Djavadian RL (2010). Neurotrophins and their receptors in early development of the mammalian nervous system. *Acta Neurobiologiae Experimentalis*.

[24] Fombonne J, Rabizadeh S, Banwait S, Mehlen P, Bredesen DE (2009). Selective vulnerability in Alzheimer's disease: amyloid precursor protein and p75NTR interaction. *Annals of Neurology*.

[25] Singh KK, Park KJ, Hong EJ (2008). Developmental axon pruning mediated by BDNF-p75NTR-dependent axon degeneration. *Nature Neuroscience*.

[26] Ganeshan V, Schor N (2011). The role of p75NTR and its signaling pathways in fenretinide-induced apoptosis in neuroblastoma cells. *Neurology*.

